# Antipsychotic medication and the elderly

**DOI:** 10.1192/j.eurpsy.2021.994

**Published:** 2021-08-13

**Authors:** D. Jardak, S. Omri, R. Feki, N. Smaoui, J. Ben Thabet, L. Zouari, N. Charfi, M. Maalej Bouali, M. Maalej

**Affiliations:** 1 Psychiatrie C, Hedi chaker hospital, sfax, Tunisia; 2 Psychiatry C Department, Hedi chaker University hospital, sfax, Tunisia

## Abstract

**Introduction:**

In recent years, the use of antipsychotics (AP) has been widely debated for reasons concerning their efficacy and safety in the elderly.

**Objectives:**

We aimed to assess the prescription of AP in the elderly subjects.

**Methods:**

We led a retrospective and descriptive study. We extracted all patients aged 65 years or older who consulted the psychiatric outpatient unit at the Hedi Chaker hospital in Sfax –Tunisia between January 1 and December 31 2019 and who were treated with AP. General, clinical and therapeutic data were collected from medical records.

**Results:**

The mean age of patients was 71,7 years. Medical conditions were observed in 53,1% of them. The reasons for consultation were behavioral disturbances (34,4%), insomnia (18,8%) and memory impairment (15,6%). The main retained diagnoses were dementia (40,6%), mood disorders (28,1%), delusional disorder (15,6%). The indications for prescribing antipsychotics were disruptive behavior (59,4%) and delirium/hallucinations (34,4%). Laboratory examinations and electrocardiogram were performed respectively in 46,8% and 15,6% of cases. AP treatment was prescribed in 90,6% of cases right from the first consultation. Atypical AP were prescribed in 56,2% of cases. Adverse effects were noted in 18,7% of patients. The average time to get a response was 7.3 weeks

**Conclusions:**

The use of AP in the elderly requires an individual assessment, case by case; particular caution is recommended.

**Conflict of interest:**

No significant relationships.

